# KIF13A—A Key Regulator of Recycling Endosome Dynamics

**DOI:** 10.3389/fcell.2022.877532

**Published:** 2022-04-25

**Authors:** Jerrin Mathew Thankachan, Subba Rao Gangi Setty

**Affiliations:** Department of Microbiology and Cell Biology, Indian Institute of Science, Bangalore, India

**Keywords:** KIF13A, kinesin-3 family, Rab22A, BLOC-1, melanosome, recycling endosome

## Abstract

Molecular motors of the kinesin superfamily (KIF) are a class of ATP-dependent motor proteins that transport cargo, including vesicles, along the tracks of the microtubule network. Around 45 KIF proteins have been described and are grouped into 14 subfamilies based on the sequence homology and domain organization. These motors facilitate a plethora of cellular functions such as vesicle transport, cell division and reorganization of the microtubule cytoskeleton. Current studies suggest that KIF13A, a kinesin-3 family member, associates with recycling endosomes and regulates their membrane dynamics (length and number). KIF13A has been implicated in several processes in many cell types, including cargo transport, recycling endosomal tubule biogenesis, cell polarity, migration and cytokinesis. Here we describe the recent advances in understanding the regulatory aspects of KIF13A motor in controlling the endosomal dynamics in addition to its structure, mechanism of its association to the membranes, regulators of motor activity, cell type-specific cargo/membrane transport, methods to measure its activity and its association with disease. Thus, this review article will provide our current understanding of the cell biological roles of KIF13A in regulating endosomal membrane remodeling.

## 1 Highlights

1) KIF13A localizes to a variety of tubular endosomes and mediates cargo recycling and transport to maturing melanosomes
2) KIF13A forms homodimers or heterodimers with KIF13B on early/sorting endosomes and generates recycling endosomes
3) KIF13A motor activity modulates the membrane dynamics (length and number) of recycling endosomes
4) KIF13A dimerization and activity requires Rab22A and regulates recycling endosome biogenesis

## 2 Introduction

Intracellular trafficking of cargo in all eukaryotic cells is powered by protein machines called molecular motors (Schliwa and Woehlke, 2003; Sweeney and Holzbaur, 2018). These mechanical units utilize chemical energy via hydrolysis of ATP for each step of their motion (Kolomeisky and Fisher, 2007). The cytoskeletal network acts as a path for these motors inside the cell and are broadly classified based on the type of tracks that these molecules move on. Microtubule-based motors are of two types, dynein and kinesin family motors. These two different motor types are primarily distinguished based on the directionality of their motion on microtubule tracks, which are organized from cell center (minus end) to periphery (plus end). Fundamentally, the dynein motors transport the cargo towards the minus-end of microtubules, and a majority of kinesins mediate the cargo transport towards the plus end. Similarly, the cargo transport on the actin cytoskeleton is mediated by myosin family motors, mainly at the cell periphery (Schliwa and Woehlke, 2003; Miki et al., 2005; Kolomeisky and Fisher, 2007; Hirokawa et al., 2009; Wang et al., 2015; Sweeney and Holzbaur, 2018; Li and Toyabe, 2020). Together, the cytoskeleton dependent motors drive diverse cargo all around the cell, required for several essential cellular processes, including vesicular transport and cell division.

The kinesin superfamily (referred to here as KIF) consists of a large number of motors and are classified into multiple families based on their differences in structure (Nakagawa et al., 1997; Miki et al., 2005; Wang et al., 2015). These members possess ATPase activity and utilize ATP to facilitate the cargo movement on microtubule tracks. In general, all kinesins have a head (motor) domain followed by the stalk (coiled-coil, CC) domain and the tail domain (Miki et al., 2005; Wang et al., 2015; Sweeney and Holzbaur, 2018). The motor domain binds to microtubules in addition to ATP, the stalk domain possess regulatory activity (provides the structural and conformational changes to the motor), and the tail domain is attached to the cargo (protein complexes/vesicles/organelles). The directional movement of the cargo bound kinesin molecule is powered by the ATPase activity of the motor domain, which is coupled with conformational changes in the kinesin molecule (Miki et al., 2005; Hirokawa et al., 2009; Verhey et al., 2011; Wang et al., 2015). Depending on the relative position of the motor domain within the sequence, the kinesin motors are classified as N-kinesins (motor domain exists at or nearer to N-terminus), C-kinesins (motor domain is observed at or nearer to C-terminus) or M-kinesins (motor domain is located internally and flanked by CC domains on either side). Based on the sequence homology of the motor domain and its relative position within the sequence, the kinesins have been reclassified into 14 families, which include kinesin-1 to kinesin-14 (Miki et al., 2005; Hirokawa et al., 2009; Verhey et al., 2011; Wang et al., 2015; Sweeney and Holzbaur, 2018).

The kinesin-3 family represents one of the largest groups in the kinesin superfamily and includes KIF1, KIF13, KIF14, KIF16 and KIF28 subfamilies. This family is distinguished from other kinesin families by virtue of structural differences in the neck region and the tail region. Further, the tail region of kinesin-3 family members contains a forkhead-associated (FHA) domain (Siddiqui and Straube, 2017). The presence of K-loop, a lysine-rich stretch in the motor domain, is also a prominent feature of kinesin-3 motors (Arpag et al., 2019). Studies have shown that the K-loop of KIF13B enhances the efficiency of its binding to ADP-bound microtubules (Soppina and Verhey, 2014). Notably, kinesin-3 members are highly processive motors that can take many steps before dissociating from the microtubule lattice, a distinct characteristic of a fast transporter (Soppina and Verhey, 2014; Arpag et al., 2019). Kinesin-3 family members transport a variety of cargoes such as organelles, vesicles and viruses, which predominantly aid their long-distance transport within the cell. Moreover, kinesin-3 family motors are also involved in other cellular functions such as cell division, viral particle trafficking, and development (Siddiqui and Straube, 2017). In this review, we have focused our discussion on one of the kinesin-3 family members, KIF13A, which has a unique role in organelle dynamics.

## 3 The Structural View of the KIF13A Motor

**FIGURE 1:**
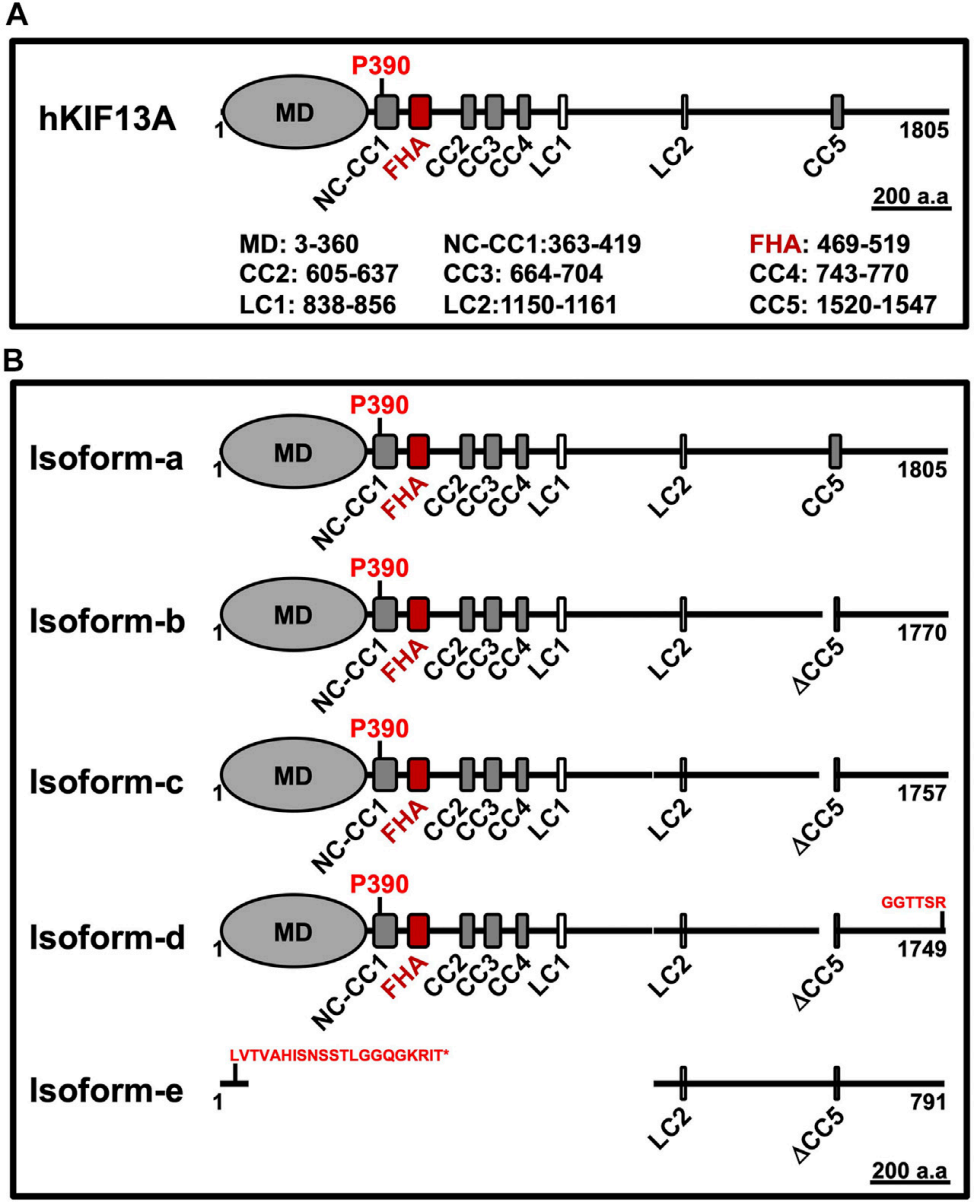
**A)** Schematic representation of KIF13A domain structure. The longest monomeric human isoform contains one motor domain (MD), one forkhead-associated (FHA) domain, five coiled-coil (CC) domains and two low complexity (LC) regions. After the MD, the neck coil (NC) region is merged with the CC1 domain. The proline (P390) residue is present in the NC-CC1 region and highlighted in the image. The length of each domain (according to the SMART tool) is indicated separately. **(B)** Schematic representation of KIF13A isoforms from the human. The five isoforms (a to e) of human KIF13A majorly differ by having different deletions, as listed in Table 1. The longest monomeric isoform-a is considered a functional form of KIF13A and used for expression studies. The change in amino acid (aa) sequence (red color letters) in the respective isoform is indicated. The isoform-e express only N-terminal 70 aa and is indicated with * after the modified sequence. The images are drawn at the displayed scale bar.

KIF13A is classified as an N-kinesin and expressed ubiquitously in all eukaryotes (Nakagawa et al., 1997). This motor was initially identified in mouse tissue by cDNA analysis (Nakagawa et al., 1997) and is evolutionarily conserved from *Caenorhabditis elegans* to mammals (Nakagawa et al., 2000; Jamain et al., 2001). KIF13A exists in five different isoforms in humans, and the isoform-a forms the longest protein having 1805 amino acids (aa) (Table 1 and Figure 1). Typically, human KIF13A (hKIF13A) is organized into three distinct domains: head or motor, stalk and tail, similar to other N-kinesins (Figure 1). The stalk domain is further divided into a neck coil (NC) domain, FHA domain, five non-continuous coiled-coil (CC) domains and two low complexity regions (Figure 1) (Soppina et al., 2014; Ren et al., 2016). Like other N-kinesins, the N-terminus contains a motor domain, which interacts with microtubules and the C-terminal tail domain associates with the cargo. Functionally, KIF13A forms homodimer *in vivo* (described below) and the monomeric motors are inactive (Patel et al., 2021). KIF13B, a homolog of KIF13A, is expressed ubiquitously and can form a heterodimer with KIF13A (Etoh and Fukuda, 2019). Structurally, KIF13B differs from KIF13A by the presence of Cap-Gly domain at the C-terminus (Mills et al., 2019). A distinct feature of KIF13 motors is the presence of a proline residue between the NC and CC regions of KIF13A (P390) and KIF13B (P391), which plays a crucial role in regulating motor function (discussed below) (Soppina et al., 2014; Patel et al., 2021).

**TABLE 1:** List of KIF13A isoforms from human.

hKIF13A isoform	NCBI ID	Length (aa)	Differences with isoform-a	Proline position	Mass (Da)
Isoform-a	NP_071396.4	1805	Canonical form	P390	202,308
Isoform-b	NP_001099036.1	1770	Deletion of aa: 1,493–1,527	P390	198,627
Isoform-c	NP_001099037.1	1757	Deletion of aa: 1,071–1,083 and 1,493-1,527	P390	197,085
Isoform-d	NP_001099038.1	1749	Deletion of aa: 1,071–1,083, 1,493–1,527 and 1,798–1,805. Change in aa: 1,792–1,797 (VIIPEA to GGTTSR)	P390	196,070
Isoform-e	NP_001230352.1 (express 1–70 aa)	70	Deletion of aa: 71–1085. Change in aa: 50–70 (KPPKVFAFDYCFWSMDESNTT to LVTVAHISNSSTLGGQGKRIT)	-	7,726

Human KIF13A isoform-a is the longest and has been widely used for research studies. Several other studies used mouse KIF13A (1750 aa), which is shorter than hKIF13A.

## 4 Basic Aspects of KIF13A Motor

### 4.1 The Localization of KIF13A and its Association With Cargo

KIF13A has been characterized as a kinesin of recycling endosomes (REs), primarily based on its association with Rab11A (Table 2) (Delevoye et al., 2014). Moreover, KIF13A has also been found on transferrin receptor (TfR)-positive vesicles (Jenkins et al., 2012). REs are part of the endocytic network and dynamically receive specific cargo from early/sorting endosomes and then recycle it to the cell surface (Goldenring, 2015; Naslavsky and Caplan, 2018). Intracellularly, the recycling of cargo operates in two ways—the fast and slow recycling pathway (Goldenring, 2015; Cullen and Steinberg, 2018). The cargo sorting to slow recycling pathway from early/sorting endosomes has been shown to mediated by clathrin adaptor protein (AP)-1 (Nakagawa et al., 2000; Delevoye et al., 2009; Sitaram et al., 2012) or recently discovered “retriever” complex (McNally et al., 2017) or FERARI complex (Solinger et al., 2020). Further, the AP-1-dependent REs are driven by KIF13A motors (Nakagawa et al., 2000; Delevoye et al., 2009). In fact, the generation or biogenesis of RE tubular structures from early/sorting endosomes are primarily dependent on KIF13A activity (Delevoye et al., 2014). Overexpression of fluorescently tagged KIF13A (KIF13A-YFP/GFP) in HeLa cells decorates a large number of long tubular structures in addition to punctate structures (referred to as endosomes) (Delevoye et al., 2014; Shakya et al., 2018; Patel et al., 2021). These longer tubular structures spread from the perinuclear region to cortical regions of the cell surface. Initial studies have shown that these tubular structures are positive for internalized transferrin and Rab11A GTPase (Delevoye et al., 2014). Rab GTPases are small GTP-binding molecules that are master regulators of membrane traffic and organelle biogenesis (Zerial and McBride, 2001; Stenmark, 2009; Zhen and Stenmark, 2015). In the GTP bound form, Rabs associate with specific vesicles/membranes and facilitate the downstream signaling or vesicle transport by recruiting respective effectors. Recent studies demonstrated that KIF13A-positive tubular structures are localized with other endosomal Rabs, Rab22A and Rab10 (Shakya et al., 2018; Etoh and Fukuda, 2019). Interestingly, these Rab GTPases are also localized to long tubular structures similar to KIF13A tubules and shown to affect the KIF13A-positive tubules upon their depletion (Delevoye et al., 2014; Shakya et al., 2018; Etoh and Fukuda, 2019; Patel et al., 2021). Additionally, a set of long tubular recycling endosomes (TREs) carrying recycling cargo are known to positive for MICAL-L1 (MICAL-like protein 1) (Sharma et al., 2009). Rab10 but not Rab8A has been shown recently to regulate MICAL-L1 tubules through KIF13A and KIF13B motors (Etoh and Fukuda, 2019). Overall, these slow recycling pathway components display tubular morphology inside the cell and are also called tubular REs. Studies have also shown that these REs primarily recycle the internalized cargo of clathrin-dependent/-independent endocytosis in pre-segregated form and biosynthetic cargo from Golgi (Xie et al., 2016). For example, TfR, a clathrin-mediated endocytosis cargo traffics to the plasma membrane (PM) through these tubular REs (De Figueiredo et al., 2001; van Dam and Stoorvogel, 2002). Further, KIF13A and KIF13B have also been shown to bind to Rab4-associated vesicles for their transport to synapse (Dey et al., 2017). As indicated, KIF13A is primarily associated with REs; however, both KIF13A and KIF13B have preferential binding to early endosomes (Bentley and Banker, 2015). The *Drosophila* KIF13A ortholog Khc-73 has been found on Rab5-associated vesicles in *Drosophila* cells (Huckaba et al., 2011). In a nutshell, endocytic compartments act as cargo for the KIF13A motor.

**TABLE 2:** List of interacting partners/regulators of KIF13.

KIF13 isoform	Interacting partner	Interacting region	Functional relevance	Regulation	References
KIF13A	Rab10	CC5 domain	Recruitment of KIF13 to endosomes	RE tubule length and number	(Etoh and Fukuda, 2019; Patel et al., 2021)
	Rab11A	Stalk and tail domains: CC3-CC4 domains	Initial stages of RE biogenesis (?)	A cohort of endosomal tubules	(Delevoye et al., 2014; Etoh and Fukuda, 2019; Patel et al., 2021)
	Rab22A	Stalk: NC-CC1 and P390	KIF13 dimerization and its processivity	RE tubule length and number	(Shakya et al., 2018; Patel et al., 2021)
	AP-1	Tail	Cargo sorting to REs	RE tubule length and number	(Nakagawa et al., 2000; Delevoye et al., 2009; Shakya et al., 2018)
	Annexin-A2	Motor, stalk, tail	Participates in the stabilization and/or scission of RE tubules (?)	RE tubule length and number	(Delevoye et al., 2016; Shakya et al., 2018)
	BLOC-1	Stalk	RE elongation and biogenesis	RE tubule length and number	Delevoye et al. (2016)
	BLOC-2	Stalk	RE elongation/tethering and biogenesis (?)	RE tubule length and number	(Dennis et al., 2015; Shakya et al., 2018)
	MICAL-L1	Possibly at CC5 through Rab10*	RE morphogenesis/biogenesis	RE tubule length and number	Etoh and Fukuda, (2019)
KIF13B	KIF13A*	dimerize	RE elongation and biogenesis	RE tubule length and number	Etoh and Fukuda, (2019)
	Rab10	CC5 domain	Recruitment of KIF13 to endosomes	RE tubule length and number	Etoh and Fukuda, (2019)

*Direct interaction between these proteins has not been reported.

“?” -requires investigation.

Kinesins generally power cargo movement towards the plus end of microtubules. In most cases, the motor interaction with cargo is direct or indirect. For example, Rab6A directly interacts with KIF20A (Echard et al., 1998). Alternatively, kinesins associate with cargo indirectly through an adaptor or linker molecule such as Rab11A interaction with KIF3B through FIP5 (Rip11) (Schonteich et al., 2008). Similarly, the protein cargo like mannose 6-phosphate receptor (M6PR) requires the clathrin adaptor AP-1 (interacts with β1-adaptin subunit) for its transport by KIF13A motor (Nakagawa et al., 2000). Interestingly, the motor-adaptor unit is essential for maintaining of RE dynamics (refers to length and number of REs) in melanocytes, which deliver the melanogenic cargoes to maturing melanosomes (Delevoye et al., 2009). Thus, the AP-1-KIF13A interaction is required for melanocyte pigmentation. Consistently, the inhibition of AP-1 interferes with the trafficking of few key molecules such as tyrosinase-related protein (TYRP1) to the maturing melanosomes, thereby leading to pigmentation defects (Delevoye et al., 2009; Campagne et al., 2018). In *C. elegans*, KIF13A mediates the anterograde trafficking of AMPA-type glutamate receptors (AMPARs) through an association with centaurin-α1 as a linker upon LTP (long-term potentiation) condition (Gutierrez et al., 2021). While many studies demonstrated the presence of an adaptor molecule working in tandem with KIF13A, the transport of serotonin type 1A receptor by KIF13A has been proposed to occur by direct binding of the motor to the cargo vesicle, in the absence of a linker molecule (Zhou et al., 2013). Overall, these studies indicate that KIF13A acts as a primary mechanical motor on the endosomal network, especially to REs.

### 4.2 Functional Aspects of KIF13A Motor in Regulating Membranes

#### 4.2.1 KIF13A Facilitates the Targeting of Cargo to the Plasma Membrane

As described above, KIF13A plays a role in transporting M6PR-containing vesicles towards the PM (Nakagawa et al., 2000). During this process, KIF13A interacts directly with the β-subunit of the AP-1 at TGN, which is independent of AP-1 interaction with clathrin (Table 2) (Nakagawa et al., 2000). Consistently, the ablation of AP-1 function using siRNA-mediated knockdown mislocalizes the KIF13A-positive endosomes towards the cell periphery (Delevoye et al., 2009). Further, overexpression of fluorescently tagged KIF13A in HeLa cells seems to stabilize the endogenous long tubular structures representing REs compared to endogenous expression (Delevoye et al., 2014; Delevoye et al., 2016; Shakya et al., 2018; Etoh and Fukuda, 2019; Patel et al., 2021). The long KIF13A-positive endogenous tubules (stained with an antibody from Bethyl laboratories, A301-077A) are seen in methanol fixed compared to formaldehyde-fixed cells (Delevoye et al., 2014). Additionally, several studies have shown the recycling of TfR to PM through KIF13A-positive REs, and this process is further regulated by multiple Rabs such as Rab11A, Rab10, and Rab22A at distinct upstream steps (Table 2) (Delevoye et al., 2014; Shakya et al., 2018; Etoh and Fukuda, 2019; Patel et al., 2021). Overall, these studies demonstrated that KIF13A mediates cargo (direct vesicles or protein molecules indirectly bound by the motor) delivery from REs and TGN to PM.

#### 4.2.2 KIF13A Regulates Melanosome Biogenesis Through REs

Melanosomes of melanocytes are grouped under a unique class of organelles called lysosome-related organelles (LROs). These cell-type specific organelles are oval shaped and filled with melanin that contributes to the pigmentation of skin, eye and hair (Raposo et al., 2007; Raposo and Marks, 2007). Melanosomes receive the cargo for their pigmentation either from endosomes or Golgi during their biogenesis that occurs in four different stages (Sitaram and Marks, 2012; Marks et al., 2013; Bowman et al., 2019; Ohbayashi and Fukuda, 2020). Several studies have shown that the pathway from endosomes, especially tubular/vesicular endosomes (representing a cohort of total REs) to maturing melanosomes, is dependent on KIF13A. This process delivers the melanin synthesizing proteins such as TYRP1, Menkes copper transporter ATP7A and oculocutaneous albinism 2 (OCA2) to stage II melanosomes, which turn into stage III and then to IV upon delivery of tyrosinase by an independent pathway (Setty et al., 2007; Setty et al., 2008; Delevoye et al., 2009; Sitaram et al., 2012; Shakya et al., 2018). Consistently, the knockdown of KIF13A or AP-1 (using siRNA) has been shown to mislocalize the above cargo, followed by degradation in the lysosomes resulting in hypopigmentation (Delevoye et al., 2009; Campagne et al., 2018). Additionally, several recent studies have shown that KIF13A regulates the biogenesis (initiation and extension) of the REs post sorting of cargo on the early/sorting endosomes (Delevoye et al., 2014; Shakya et al., 2018; Etoh and Fukuda, 2019). These studies demonstrated a direct role for KIF13A in RE biogenesis. In contrast to KIF13A homodimer’s regulation over REs, the KIF13A/B heterodimer also contributes to a similar extent; however, we predict the regulation is related to the expression levels of these motors within the cell (Etoh and Fukuda, 2019). In non-melanocytes such as HeLa cells, KIF13A has been shown to be a key regulator of TfR recycling to PM, which follows the same mechanism described for melanosome biogenesis (Delevoye et al., 2009; Delevoye et al., 2014; Shakya et al., 2018). With these results, we predict that REs may serve a dual role in melanocytes, including the trafficking of TfR to PM in addition to cargo transport to melanosomes.

#### 4.2.3 KIF13A—Crosstalk With Multiple Rab GTPases and Multi-Subunit Protein Complexes on Endosomal Membranes

Rabs play a central role in regulating motor activity, especially helping the motors associate with membranes (direct or indirectly) and cytoskeleton that facilitates the cargo transport/membrane fusion (Stenmark, 2009; Zhen and Stenmark, 2015). For example, the direct Rab-motor interactions have been reported for Rab6-KIF20A (Echard et al., 1998), Rab14-KIF16B (Ueno et al., 2011) and Rab4A with dynein (Bielli et al., 2001). The indirect Rab-motor interactions are mediated through adaptor/Rab effector molecules that interface between the motor and the Rab molecule. Among the many reported, few of them include Rab11A with KIF3B through Rip11 (Schonteich et al., 2008), Rab27a with myosin Va through melanophilin (Fukuda et al., 2002; Nagashima et al., 2002), Rab11A with dynein through Rab11-FIP3 (Horgan et al., 2010) and Rab7 with dynein-dynactin through RILP (Jordens et al., 2001). Recent studies on KIF13A have thrown a light on the layers of Rab regulation over the motor activity/function (described below) in controlling the membrane dynamics (length and number of tubular endosomes) (Delevoye et al., 2014; Shakya et al., 2018; Etoh and Fukuda, 2019; Patel et al., 2021). Thus, these observations imply that organelle transport/membrane dynamics meditated by kinesin motors is tightly controlled by multiple factors, including Rabs, which ensure the unperturbed homeostasis within the cell.

**FIGURE 2:**
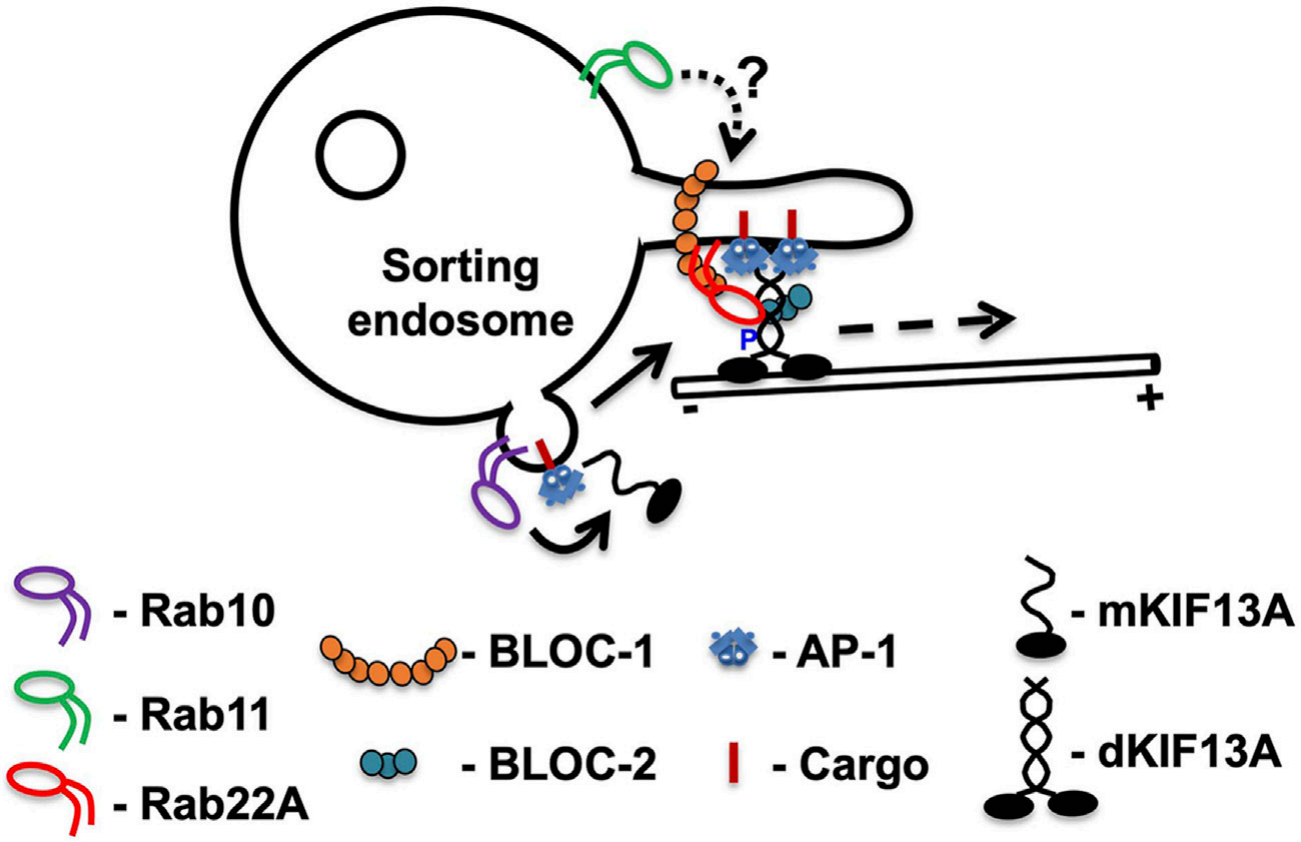
The current model of KIF13A functions in regulating RE dynamics. Based on published studies, we propose a model for KIF13A regulation on RE dynamics (length and number). Monomeric (m) KIF13As are recruited to sorting endosomes upon activation of Rab10 (physically binds to C-terminal tail of KIF13A) to cargo sorted endosomal buds (bent arrow). These monomeric KIF13A forms homodimers (d) or heterodimers by pairing with KIF13B (not shown). The P390 in the KIF13A monomers inhibits the formation of a dimer, which will be relieved by active Rab22A on endosomal buds. Rab22A interacts with KIF13A at the NC-CC1 domain and facilitates dimerization and motor activation. The tail domain of dimeric KIF13A associates with cargo through cargo adaptor AP (adaptor protein)-1, and the motor domain associates with microtubules. During this process, Rab22A recruits BLOC-1 and BLOC-2 to the endosomal bud and facilitates the elongation and extension of endosomal buds into REs with the help of KIF13A along the microtubule tracks. Note that the REs are generated due to balanced tug-of-war between dynein and KIF13A motors during the tubule extension (not shown). We hypothesize either a Rab switch (indicated as an arrow) or Rab coordination between Rab10 and Rab22A during dimerization and activation of KIF13A. The dashed arrow represents the direction of elongation towards the + end of the microtubule. We also predict that Rab11A functions as an upstream factor and regulates the endosomal maturation and initial stages of RE formation. Even though Rab11 interacts directly with KIF13A, its precise role in regulating the KIF13A function requires investigation (shown as dashed arrow with ?).

KIF13A-positive tubules are marked by Rab11A, functioning as an upstream factor and is required for endosomal maturation and RE formation (Delevoye et al., 2014). Initial studies have shown that KIF13A interacts with GTP-bound Rab11A and maintains the RE morphogenesis in HeLa cells (Table 2). Depletion of KIF13A impedes the formation of Rab11A-positive REs and generate the enlarged/vacuolar endosomes (Delevoye et al., 2014). Notably, the elongation of KIF13A-decorated REs has been shown to be controlled by a multi-subunit protein complex BLOC-1 (biogenesis of lysosome-related organelles complex-1) based on siRNA/shRNA mediated knockdown of BLOC-1 in HeLa cells (Delevoye et al., 2016; Shakya et al., 2018). The regulation predicted here is that Rab11A crosstalks with BLOC-1 and KIF13A independently (Shakya et al., 2018). Consistently, cells depleted for either BLOC-1 or Rab11A display defective KIF13A-positive tubule formation and are unable to recycle the labelled-transferrin due to their accumulation in the enlarged endosomal compartments (Delevoye et al., 2014; Shakya et al., 2018). Further, the motor domain of KIF13A is necessary for endosomal tubulation, whereas the stalk and tail domains facilitate the targeting of KIF13A to early/sorting endosomal domains (Delevoye et al., 2014; Shakya et al., 2018). The role of actin-network came into light after the disruption of actin polymerization regulators such as Arp2/3 complex and annexin A2 (AnxA2) in cells that display a defect in the formation of long KIF13A-tubular structures. Moreover, KIF13A (stalk domain) has been shown to interact with AnxA2 (Delevoye et al., 2016). These studies imply that KIF13A possibly associates with AnxA2 (actin dynamics) surrounding the endosomes on one side (tail domain) and with the microtubule network on another side (motor domain) during the elongation or for the stabilization of KIF13A-decorated REs. Additionally, in cooperation with BLOC-1, AnxA2 was proposed to regulate KIF13A-REs, required either for their stabilization and/or scission (Table 2) (Delevoye et al., 2016). At the cellular level, these processes are required for melanocyte pigmentation (Setty et al., 2007; Delevoye et al., 2016; Shakya et al., 2018). However, it is unclear how these molecules interact sequentially on the membrane. Recent studies shed light on these mechanisms and showed that another GTPase Rab22A critically plays a central role in regulating the entire process (Figure 2). Rab22A has been shown to interact directly with BLOC-1 and BLOC-2 (another three-subunit cytosolic complex) and also with KIF13A (Table 2) (Shakya et al., 2018). Moreover, Rab22A sequentially recruits BLOC-1 and BLOC-2 to the endosomal membranes that are positive for AP-1-KIF13A and facilitates the extension of endosomal buds into tubules with the help of the KIF13A motor (Shakya et al., 2018). In parallel, the work done by Etoh and Fukuda (2019) elucidated the role of KIF13A in regulating a different set of endosomal tubules labeled with MICAL-L1 (Etoh and Fukuda, 2019). These studies identified another Rab GTPase, Rab10, to regulate the formation of KIF13A-positive MICAL-L1 tubules (Table 2). Moreover, the formation of KIF13A/B heterodimers and their importance in maintaining the MICAL-L1 tubules has been highlighted (Etoh and Fukuda, 2019). However, the crosstalk between Rab10 and Rab22A in regulating KIF13-positive tubules requires future investigation. These studies have shown that Rab10 functions as an upstream factor to Rab22A-mediated RE biogenesis and is predicted to recruit KIF13A monomers to the endosomal membranes (Figure 2) (Patel et al., 2021). Consistently, Rab10 knockdown in HeLa cells mislocalized KIF13A to the cytosol (Patel et al., 2021) and was not compensated by active Rab22A (Etoh and Fukuda, 2019). In a nutshell, Rab22A has been proposed to recruit BLOC-1 and BLOC-2 to facilitate elongation of KIF13A tubules (Shakya et al., 2018) while Rab10 is predicted to recruit KIF13A/B to elongate the MICAL-L1-positive tubules (Etoh and Fukuda, 2019). We predict that Rab10 and Rab22A might share a cohort of elongated endosomal tubules in addition to the existence of specific individual tubules. It would be interesting to understand the KIF13A specificity and the membrane recruitment mechanisms to these endosomal membranes (Rab10- and/or Rab22A- positive) for generating a different set of REs. Nevertheless, a recent study demonstrated that Rab22A binds to the NC-CC domain of KIF13A, would relieve the proline-mediated inhibition of KIF13A motor function (see below) and enables the dimerization and activation of the KIF13A motor (Figure 2) (Patel et al., 2021). These studies indicated a crucial role of Rab22A in regulating the KIF13A activity and function. However, the role of Rab11A needs to be evaluated in future.

Studies have shown that several multi-subunit complexes such as BLOC-1 are known to regulate both KIF13A-positive RE biogenesis and cargo sorting on sorting endosomes (Setty et al., 2007; Setty et al., 2008; Delevoye et al., 2016). Similarly, two other complexes, namely retromer and retriever, modulate the cargo recycling from endosomes to PM (Cullen and Steinberg, 2018). Retromer comprises two subcomplexes: a heterotrimeric complex of VPS26, VPS29 and VPS35, and a dimer of SNX1 or SNX2 and SNX5, SNX6 or SNX32 (Naslavsky and Caplan, 2018). Further, the retromer complex associates with the cargo adaptor SNX27 and actin nucleation promoting complex WASH on endosomes. Studies have shown that actin nucleation via the Arp2/3 complex contributes to the elongation of RE tubules and the WASH complex in regulating the actin-dependent endosomal membrane scission (Derivery et al., 2009; Gomez and Billadeau, 2009; Gomez et al., 2012). In contrast, the WASH complex cooperates with BLOC-1 to regulate the sorting and targeting of BLOC-1-dependent cargo (Monfregola et al., 2010; Ryder et al., 2013). However, the depletion of WASH or the retromer subunit VPS35 did not affect KIF13-RE biogenesis (Derivery et al., 2009; Gomez and Billadeau, 2009), suggesting a role for WASH/retromer-independent and ARP2/3-dependent actin polymerization during the formation of KIF13A-REs. Interestingly, the trafficking of ATP7A from endosomes to melanosomes is dependent on BLOC-1 (Setty et al., 2008) and to the PM is regulated by multiple complexes Rab22A and AP-1 (Holloway et al., 2013), SNX27-retromer complex (Steinberg et al., 2013), COMMD1/CCC complex (Phillips-Krawczak et al., 2015) or WASH complex (Ryder et al., 2013). Thus, the recycling of ATP7A seems to be facilitated by multiple recycling pathways indicating crosstalk between them. The retriever is a heterotrimer complex composing VPS35L (C16orf62), VPS26C (DSCR3) and VPS29, and associates with SNX17, and couples to CCC and WASH complexes to facilitate recycling of α5β1-integrin to PM (McNally et al., 2017; Singla et al., 2019). Another complex FERARI (consists of Rab11FIP5, Rabenosyn-5, VPS45, VIPAS39, ANK3 and EHD1) has been shown to coordinate the kiss-and-run dynamics of Rab11A endosomes with SNX-1-positive REs (Solinger et al., 2020). However, the association of KIF13A to these endosomal complexes needs to be investigated further.

#### 4.2.4 KIF13A—A Role in Pathogenesis and Other Cellular Processes

Studies have shown that certain intracellular pathogens hijack the KIF13A-mediated plus-end directed trafficking for their survival and pathogenesis (Fehling et al., 2013; Ramos-Nascimento et al., 2017). Interestingly, certain viral proteins such as matrix protein Z of arenavirus use KIF13A to traffic to the PM, essential for virus production at the PM (Fehling et al., 2013). Similarly, KIF13A transports Rab11A-positive vesicles containing ribonucleoproteins of influenza A virus to the cell surface during infection (Ramos-Nascimento et al., 2017). Consistently, the reduced levels of KIF13A have a corresponding decline in virus production. These studies show that viruses can exploit KIF13A-mediated trafficking to facilitate the infection and can serve as an important target molecule in the context of viral infection.

The role of KIF13A in other basic cellular processes have also been studied. KIF13A facilitates the recycling of a small GTPase RhoB *via* Rab11A endosomes to the PM and thereby controls PM blebbing and amoeboid migration (Gong et al., 2018). Thus, KIF13A plays a role in cell migration.

The cytokines (IL-2 and IL-7) released by T lymphocytes have been shown to be regulated by the PI3K-mTORC1 signaling pathway, which in turn requires the KIF13A-mediated transport of M6PR to the cell surface, and therefore controls the fate of T-cells (Ahmed and Xiang, 2017). Interestingly, mTORC1 inhibition suppresses the AP-1-KIF13A activity leading to reduced surface expression of M6PR (Ahmed and Xiang, 2017). KIF13A-mediated transport of PI(3)P binding protein FYVE-CENT and TTC19 from the centrosome to midbody is important for cytokinesis. Correspondingly, KIF13A depletion results in cytokinesis arrest in the cells (Sagona et al., 2010), indicating a role for KIF13A in cell division.

In neuronal cells, LTP processes are found to be dependent on KIF13A activity. KIF13A facilitates the trafficking of a subset of glutamate receptors to the synapse through Rab11-FIP2 endosomes during LTP induction (Gutierrez et al., 2021). Additionally, KIF13A or KIF13B have been observed on myosin-V labelled vesicles during the neuronal vesicle transport process (Frank et al., 2020). Similarly, KIF13A and KIF13B are shown to associate with a subset of dendritic vesicles and TfR-positive vesicles in dendritic cells (Jenkins et al., 2012).

## 5 Regulatory Aspects of dKIF13A Motor

Kinesins in the cytosol exist in an autoinhibited state (without bound cargo) due to intramolecular interactions within the kinesin molecule (Verhey et al., 2011). In some kinesin-3 family motors, the intramolecular interactions between the neck and tail keep the molecule inactive and monomeric. This model of autoinhibition has been proposed for KIF13A motors, and their activation results in a conformational change that leads to dimerization (dKIF13A) of the kinesin molecules by involving intermolecular interactions between the neck and tail regions (Siddiqui and Straube, 2017). Studies have shown that cargo binding and motor dimerization are essential for the processive transport of kinesin-3 family motors (Soppina et al., 2014). In KIF13A, the NC domain facilitates the motor dimerization, and CC domains are involved in motor regulation by controlling the dimerization and processivity (Soppina et al., 2014). Interestingly, Ren et al., 2016 showed the minimal regulatory element of KIF13A as NC-CC-FHA tandem, essential for motor function (Ren et al., 2016). Alike other kinesin-3 family motors, the NC region in KIF13A is short in length to form a CC dimer during motor dimerization. Thus, the FHA domain with the short CC domain will fulfil the requirement in forming an extended dimer. Unlike other kinesin-3 family members, KIF13A and KIF13B contain a proline (P390 and P391, respectively) residue at the junction between the NC and CC1 domains. This unique proline causes the misalignment of NC and CC1 segments that restricts motor dimerization and prevents motor processivity (Soppina et al., 2014). Interestingly, the deletion of proline 390 residue in KIF13A enhances the processivity of dimeric motor due to the formation of extended CC dimer by the contribution of NC-CC-FHA domains (Soppina et al., 2014; Ren et al., 2016). However, the interaction between the NC and CC1 segments is sufficient for motor dimerization in other kinesin-3 family motors like KIF1A. Thus, the regulation of proline-dependent motor dimerization is unique to KIF13 (A or B) motors. Nevertheless, the *in vivo* mechanism/s behind this regulation was not clear until recently. Studies by (Shakya et al., 2018) showed that small GTPase Rab22A interacts with the stalk domain of KIF13A (Table 2). In contrast, Rab11A was also shown to interact with the tail and stalk domains of KIF13A (Table 2); however, Rab11A could not rescue the defective KIF13A-positive REs observed in Rab22A knockdown cells (Shakya et al., 2018). Very recently, the work done by (Patel et al., 2021), uncovered the *in vivo* mechanism that relieves the proline-mediated inhibition of KIF13A dimerization (Patel et al., 2021). Surprisingly, the Rab22A interaction with KIF13A (initially shown by (Shakya et al., 2018)), especially at the NC-CC1 region, relieved the proline-induced steric hindrance, enabling the motor to dimerize, which then undergoes processive motion (Figure 2 and Table 2) (Patel et al., 2021). Consistently, the ΔP390 mutant of KIF13A was able to form an extended dimer by utilizing NC-CC1-FHA domains, which resulted in functional motor (Soppina et al., 2014; Ren et al., 2016). Additionally, the ΔP390 mutant displayed transport with higher velocity and generated higher force than wild type KIF13A, resulting in defective RE formation (Patel et al., 2021). Overall, these studies demonstrated an *in vivo* role for Rab22A in controlling the KIF13A dimerization and motor processivity by binding to P390 residue at NC-CC1 domains.

## 6 Methods to Measure the KIF13 Activity

The membrane dynamics mediated by the dKIF13A motor are measured as a parameter of tubule length and number in a given condition. This will be measured by overexpressing of epitope-tagged (GFP/YFP at C-terminus) KIF13A in cells. The image analysis protocol for these measurements has been developed on the Fiji platform and described previously in (Delevoye et al., 2016; Shakya et al., 2018). Note that the length of each branch and number of branches of a skeletonized image (see step 2 of the protocol) corresponds to length and number of KIF13A-labeled REs.

The following Macro has been adapted from (Shakya et al., 2018) to measure the RE tubule length and number in a given cell:

1) Cell expressing KIF13A-GFP/YFP are fixed with formaldehyde or ice-cold methanol on a glass coverslip and then imaged under the fluorescence microscope.
2) The following step wise workflow can be used for image processing and the analysis of KIF13A-positive endosomal tubules using Fiji
  a) Open the image in Fiji.
  b) Separate the channels for multichannel image using the *Split channels* option in the *Image* tab: *Image*→*Color*→*Split channels*.
  c) Select the best focussed image planes containing KIF13A-positive tubules and obtain a maximum intensity projection (MIP) of the image using *Image*→*Stacks*→*Z Project*.
  d) Process the MIP image as follows:
    - Convert into an 8-bit image: *Image*→*Type*→*8 bit*.
    - In the *Plugins* tab, go to *Analyze*, then select the “*Tubeness*” option: *Analyze*→*Tubeness*.
    - In the *Tubeness* window, set the appropriate sigma value for tubeness (the recorded Macro uses 0.1935).
    - Adjust image threshold by setting the appropriate image threshold value: *Image*→*Threshold*.
    - Convert the image to its binary format: *Process*→*Binary*→*Convert to Mask*.
    - Skeletonize the image: Process→Binary→Skeletonize.
    - Analyze the skeleton: Analyze→Skeleton→Analyze Skeleton (2D/3D).
    - Select the “*Average branch length*” column values in the *Results* window.
    - Sort the values in the desired size range (the recorded Macro uses 1.3—20 µm).

Note: The steps described in (d) can be automated and recorded by writing a Macro similar to described below. The Macro flow can be processed using the *Macros* option in the *Plugins* tab.

3) The following recorded *Macro programme* can be used directly to measure the parameters. The below link will guide the user to learn and modify the *Macro programme* on Fiji platform: https://imagej.nih.gov/ij/developer/macro/macros.html run(“8-bit”); run(“Tubeness”, “sigma = 0.1935 use”); run(“8-bit”); setAutoThreshold(“Default dark”); //run (“Threshold...”); //setThreshold(40, 255); setOption(“BlackBackground”,false); run(“Convert to Mask”); and run(“Skeletonize”).

Note: Select the tubules of size range between 1.3—20 μm (more than the average diameter of endosomes) for the calculations. The user can decide variations on the size range.

The following parameters require optimization by the user to measure any type of tubular structures depending on their experimental conditions:

1) Sigma value for the tubeness with a notation that high sigma value for thicker tubule.
2) Threshold values to be optimized by preserving the tubules’ integrity with a lower background intensity
3) The size range for tubule length should be chosen more than the average diameter of endosomes in that cell type

Nevertheless, the above method can be used to measure the dynamics of any tubules such as Rab22A/Rab11/Rab10 -positive REs or MICAL-L1-positive REs.

## 7 Disease Links to KIF13A Motor

KIF13A was first identified at a locus with suspected linkage to schizophrenia (Jamain et al., 2001). Since endocytic recycling is essential for cellular homeostasis, defects therein can lead to maladies. Dysfunction of KIF13A has been implicated in many cancers (Gong et al., 2018; Zhang et al., 2018; Banerjee et al., 2021), and its enhanced amplification is observed in some cancers (Chandrasekaran et al., 2015). A KIF13A fusion with RET sequences encoding tyrosine kinase (KIF13-RET) has been reported in lung adenocarcinoma (Zhang et al., 2018). Further, enhanced expression of KIF13A has been reported in retinoblastomas (Chen et al., 2002; Orlic et al., 2006). A study describing ovarian tumor samples showed a splice site mutation in KIF13A (Lee et al., 2015). Interestingly, KIF13A was observed to mislocalize from the Golgi region to the nucleus during glioma progression (Lee et al., 2013). Transcriptional regulation of KIF13A by epithelial-to-mesenchymal transcription factors has been found to promote trafficking through endosomal recycling, which facilitates the establishment of a polarity axis during cell migration and has been found to be essential for cancer progression in lung adenocarcinoma cells (Banerjee et al., 2021). In neuronal cells, the absence of KIF13A causes reduced cell surface expression of a set of serotonin receptors, leading to elevated anxiety phenotype in KIF13A knockout mice (Zhou et al., 2013). In a different context, few of the viruses (notably the influenza virus and Lassa virus) utilize the KIF13A motor for intracellular transport of viral proteins (Ramos-Nascimento et al., 2017), which causes viral hemorrhagic fever (Fehling et al., 2013). Thus, the dysregulation of KIF13A function has been implicated in many diseases.

## 8 Future Prospective

Enhanced understanding of organelle dynamics in a recent time has made an impact on our knowledge of different cellular processes. In addition, the change in number and/or size of eukaryotic organelles play an important role in intracellular signaling and their associated cellular properties, observed in multiple disease conditions (refer (Makhoul et al., 2019) for Golgi; (Ballabio and Bonifacino, 2020) for lysosome; (Delevoye et al., 2019) for LROs) or link to monogenetic disorders (Olkkonen and Ikonen, 2006; Platt et al., 2018). Similarly, the defects in the formation of REs (like in BLOC-1 deficient melanocytes) results in Hermansky-Pudlak syndrome (Wei and Li, 2013; Bowman et al., 2019). In contrast, the RE biogenesis defects in non-melanocytes such as HeLa does not show any significant disease phenotypes (due to unknown compensatory mechanisms) other than reduced recycling of cargo from endosomes to the PM. Interestingly, the RE dynamics has been shown to be altered in many cancers. Although several studies have reported the regulators of RE biogenesis, the sequential mechanism of their formation is not well understood. Based on the current literature, we propose the following model for RE biogenesis/dynamics (Figure 2): (a) post sorting of cargo by clathrin adaptors (especially AP-1) generate endosomal buds on the sorting endosomes; (b) we predict that activated Rab10 on these endosomal buds recruits an inactive monomeric KIF13A (mKIF13A) to the membranes; (c) binding of Rab22A to KIF13A (at NC-CC1 domain) relieves the inhibition mediated by P390 and forms KIF13A dimers (dKIF13A), which interacts with the cargo bound AP-1 with its C-terminus and motor domains associate with the microtubules; (d) in parallel, Rab22A recruits BLOC-1 and BLOC-2 on to these membranes and facilitate the extension of buds into tubules with the help of dKIF13A motor. Thus, these processes result in the formation of long tubular endosomes (representing REs), which undergo fission followed by fusion with PM or with maturing melanosomes in melanocytes. This model requires extensive cellular analysis of different types of REs and their crosstalk with the known machinery. We hypothesize that Rab11A acts upstream to this pathway, and its precise role needs to be evaluated. Although, the role of the actin cytoskeleton in regulating the endosomal dynamics/stability/fission have been described, the critical role in KIF13A-generated REs needs to be addressed in future. Hence, studying the crosstalk between different Rabs and the RE types may provide a preliminary clue to the RE biogenesis.
